# Deep Brain Stimulator Infection by Oligella: A Case Report and Review of the Literature

**DOI:** 10.7759/cureus.35133

**Published:** 2023-02-18

**Authors:** Matthew K Edwards, Vidya Kollu, Gautam S Kalyatanda

**Affiliations:** 1 Department of Infectious Diseases and Global Medicine, Case Western Reserve University School of Medicine, Cleveland, USA; 2 Department of Infectious Diseases and Global Medicine, University of Florida College of Medicine, Gainesville, USA

**Keywords:** device-associated infection, tourette syndrome, oligella urethralis, deep brain stimulator, oligella ureolytica

## Abstract

*Oligella* is a commensal bacteria genus of the human urinary tract that rarely precipitates clinical infections. We report the case of an asymptomatic 24-year-old male with a medical history of Tourette syndrome and the recent placement of deep brain stimulator leads, which were found to be co-infected with *Oligella* species during hardware implantation. This is the first reported case of a deep brain stimulator infection by *Oligella*, a potentially under-recognized and emerging opportunistic bacteria. We review the previously published cases of extra-genitourinary *Oligella* infections and detail the clinical management of this uncommon pathogen.

## Introduction

*Oligella* is a genus of aerobic Gram-negative coccobacilli comprising two species, *O. ureolytica* (formerly known as CDC group IVe) and *O. urethralis* (formerly known as *Moraxella urethralis*) [1]. These bacteria are particularly difficult to isolate using conventional laboratory techniques and knowledge of their antimicrobial susceptibility is limited [2]. Available data suggest that *O. ureolytica* are susceptible to far fewer antimicrobials than *O. urethralis*, yet both are generally susceptible to aminoglycosides and trimethoprim-sulfamethoxazole [2]. There is seldom cause to isolate *Oligella* species (spp.), as they are normally commensal within the human urinary tract and rarely precipitate clinical infections [2]. Pathogenic *Oligella* spp. are mostly encountered in the genitourinary tract, but both species have been implicated in respiratory infections of patients with cystic fibrosis, suggesting an additional tropism for the lower pulmonary tract [2]. Few other cases of extra-genitourinary infections by *Oligella* spp. have been reported in the literature and have included infections of the blood, lymphatics, and synovia [2-12]. Infections with *Oligella* spp. typically arise in patients with underlying medical conditions, particularly those associated with immunosuppression. Despite their low virulence, *Oligella* spp. may represent an under-recognized and emerging opportunistic pathogen [5]. Here we examine the first reported case of a deep brain stimulator (DBS) infection by *Oligella* spp. and provide a review of the literature on *Oligella* infections.

## Case presentation

A 24-year-old male with a past medical history of Tourette syndrome (diagnosed at the age of five years), depression, attention deficit hyperactive disorder, and obsessive-compulsive disorder were evaluated for the second procedure of elective DBS surgery. He was deemed a candidate for DBS placement because of uncontrolled tics of the bilateral upper extremities and head refractory to maximal medical management. In the first procedure, he underwent bilateral DBS leads implantation five weeks before the current admission, during which he was scheduled to have both leads connected to a pulse generator in the chest. This two-step process is the routine approach to DBS implantation. The patient denied any recent fever, chills or rigors, nausea, vomiting, or diarrhea, as well as any redness, pain, swelling, or drainage from the surgical site. A blood panel performed prior to surgery was notable for a white blood cell count of 11.0 x 10^9^ cells/L (reference: 4-11 x 10^9^ cells/L) and normal differential. During the procedure, the surgeon observed a significant amount of gelatinous material surrounding the connectors and harvested samples for a Gram stain. The stain showed Gram-positive cocci. Therefore, the hardware implantation procedure was aborted and the infected left-sided DBS hardware was explanted and sent for fungal, aerobic and anaerobic bacterial, and acid-fast bacilli cultures.

Broad-spectrum antimicrobials were initiated, including 750mg intravenous (IV) vancomycin every 12 hours, 2g IV cefepime every 12 hours, and 500mg IV metronidazole every eight hours. The aerobic cultures grew *Corynebacterium* spp., *Oligella* spp., and coagulase-negative *Staphylococcus* (CoNS) spp. Our laboratory does not perform the sensitivities for *Corynebacterium* spp. and so only the sensitivities for *Oligella* and CoNS are reported (Table 1). Unfortunately, due to temporary limitations of our laboratory, we were unable to speciate the cultured *Oligella.*

**Table 1:** Sensitivity of Oligella and coagulate-negative Staphylococcus species determined by Kirby-Bauer test No Clinical and Laboratory Standards Institute (CLSI) interpretation is available for this drug-organism combination. P-T: Piperacillin/Tazobactam; T-S: Trimethoprim/Sulfamethoxazole; S: Susceptible; R: Resistant; I: Intermediate

Antimicrobials	*Oligella* spp. Susceptibility	Antimicrobials	CoNS Susceptibility
Amikacin	S	Ciprofloxacin	S
Ampicillin	S	Erythromycin	R
Aztreonam	S	Gentamicin	S
Cefepime	S	Levofloxacin	S
Cefoxitin	S	Linezolid	S
Ceftazidime	S	Minocycline	S
Ciprofloxacin	R	Moxifloxacin	S
Clindamycin	R	Oxacillin	R
Erythromycin	I	Penicillin	R
Gentamicin	S	Rifampin	S
Levofloxacin	R	Tetracycline	S
Linezolid	R	T-S	S
Meropenem	S	Vancomycin	S
P-T	S		
Rifampin	R		
Tetracycline	S		
Tobramycin	S		
T-S	S		

The patient admitted to vaping and occasional alcohol use but denied any history of illicit drug use. He suffered from tics and stated that if these occurred during micturition, urine would splatter and even reach his head. He further noted that he had not been applying surgical wound dressings since his initial DBS surgery. Once the sensitivities were finalized, the antimicrobials were narrowed to IV vancomycin and IV cefepime, which were continued for a total of six weeks following DBS lead removal. The patient did not report any side effects of treatment and vancomycin serum trough concentrations were monitored to maintain a level of 15-20mcg/mL. After the completion of antimicrobial therapy, the patient underwent reimplantation of the DBS lead and is now awaiting pulse generator placement.

## Discussion

Tourette syndrome is a neuropsychiatric condition characterized by repetitive and non-rhythmic movements and vocalizations resulting from dysfunction in a basal ganglia-thalamo-cortical circuit [13]. In certain cases, these tics may be debilitating and resistant to medication, prompting the investigational use of DBSs [14]. The DBS is an implanted device consisting of a subcutaneous pulse generator, an extension wire, and an intracranial lead, which delivers electrical stimulations to target regions of the brain to treat movement disorders including Parkinson’s disease, dystonia, and essential tremor [14]. As the clinical use of DBS increases, so does concern for infection of these devices with relatively common pathogens [15]. Estimates of DBS infection rates vary from 1% to 23%, with such a large range attributable to varying practices of antibiotic prophylaxis, surgical technique, and surveillance [15,16]. Infections may involve each of the three components of the DBS, though pulse generator infections occur most frequently [16].

DBS infections typically arise from common skin commensals, including *Staphylococcus aureus*, *S. epidermidis*, *Pseudomonas aeruginosa*, and *Propionibacterium acnes* [16]. Instances of DBS infections by atypical bacteria that are not skin commensals have been reported in the literature. However, in almost all cases, these have been with organisms common to the environment and have easily understood mechanisms of infection [16,17]. In this case, we found an intracranial lead infected by a combination of common skin flora (CoNS and Corynebacteria) and *Oligella*, an organism predominately found in the human urinary tract. Because the patient denied the use of surgical wound packing and reported occasional splashing of his head during micturition, we posit that a feasible source of infection was the patient’s own urine.

A review of the literature yielded only 12 prior reported cases of infection by either *Oligella* spp. (Table 2). In nearly all cases, infections occurred in patients with an identified source of immunosuppression, including malignancy, AIDS, and newborn status. Obstruction of the urinary tract also appeared to be a predisposing factor for *Oligella* bacteremia. In two cases (both infections with *O. ureolytica*), patients had wounds thought to be the nidus of infection, including one elderly woman who had fallen and spent four days laying in her own urine [4,5]. *Oligella* infections may be more common than reported, as many laboratories do not pursue the identification of uncommon bacteria to the genus level and few incubate cultures for the four days required by *Oligella* spp. [5].

**Table 2:** Published cases of extra-genitourinary Oligella spp. infections Certain published cases were excluded as likely contaminants or not fitting typical laboratory profiles for *Oligella* spp.

Year	Age/Sex	Bacterium	Culture Source	Concurrent Conditions	Reference
2023	24/M	*Oligella* spp.	Hardware	Tourette syndrome, deep brain stimulator	
2019	51/M	O. urethralis	Protected bronchial specimen	Pulmonary abscess, non-small lung cancer	(2)
2017	90/F	O. urethralis	Blood	Diabetes mellitus	(3)
2016	66/M	O. ureolytica	Blood	Aortic valve bio-prosthesis	(4)
2015	66/F	O. ureolytica	Blood	Contaminated wound, sepsis	(5)
2014	30/M	O. ureolytica	Blood	Metastatic lung adenocarcinoma	(6)
2013	Newborn/F	O. ureolytica	Blood	None	(7)
1996	69/M	O. urethralis	Peritoneal dialysate	Chronic ambulatory peritoneal dialysis, diabetes mellitus	(8)
1996	29/M	O. urethralis	Peritoneal dialysate	Chronic ambulatory peritoneal dialysis	(8)
1995	49/F	O. ureolytica	Cervical lymph node	Non-Hodgkin lymphoma	(9)
1993	75/M	O. urethralis	Blood	Metastatic colorectal cancer, obstructive uropathy	(10)
1993	40/M	O. ureolytica	Blood	AIDS, sacral ulcer, diarrhea	(11)
1992	83/M	O. urethralis	Knee fluid	Rectal adenocarcinoma, septic arthritis	(12)

Approaches to managing DBS infections are variable and depend upon the extent of the infection [17]. Initial empiric antibiotic therapy should cover commonly encountered pathogens, while treatment after susceptibility determination should target the identified organisms [16]. Antibiotic therapy is infrequently sufficient for treating DBS infections, and its combination with partial or complete removal of hardware is often required [17]. Complete system removal is necessary when there is evidence of intracranial lead infection [16]. In this case, with a Gram stain of gelatinous material surrounding the connector site showing Gram-positive cocci, the entire system was explanted and the patient was started on broad-spectrum IV antibiotics. The ultimate antibiotic choice was determined by sensitivity testing and the treatment duration of six weeks was chosen based on the typical approach to managing cerebritis. Without a complete device removal, however, the patient would have likely experienced recurrent infections following antibiotic treatments.

## Conclusions

While the proposed mechanism of infection in the current case is extremely unlikely for most patients receiving DBS implantation, we believe that this case highlights the vulnerability of these systems to relatively avirulent organisms, as well as the growing recognition of *Oligella* as a source of opportunistic infection. Providers should appreciate the unusual causes of DBS infection and should consider *Oligella* as a potential pathogen when cultures are negative for common skin flora. Reporting novel cases of infection may be valuable for guiding provider decisions on antimicrobial treatment choices and methods of administration.
